# Analysis of DGAT2 mutations reveals potential links between cancer and lipid droplet deregulation

**DOI:** 10.1093/geroni/igab046.2530

**Published:** 2021-12-17

**Authors:** Meghan Graber, Hayley Barta

**Affiliations:** Xavier University, Cincinnati, Ohio, United States

## Abstract

Diacylglycerol O-acyltransferase 2 is a transmembrane protein encoded by the DGAT2 gene that functions in lipid metabolism, triacylglycerol synthesis, and lipid droplet regulation. Since cancer cells exhibit altered lipid metabolism, it has been proposed that mutations in DGAT2 may contribute to this state. Using data from the Catalogue of Somatic Mutations in Cancer (COSMIC), we analyzed all reported DGAT2 mutations in human cancers. Bioinformatics analyses were performed to highlight the connections between age, pathogenicity, and cancer tissue type. Mutations are generally associated with samples from older individuals, except for those in glioblastomas which occur earlier. We also found that several DGAT2 mutations fall within the catalytic site of the enzyme and may affect enzyme function. Thus, these mutations may contribute to altered cancer metabolism. We identified D222V as a mutation hotspot neighboring a previously discovered Y223H mutation that causes Axonal Charcot-Marie-Tooth disease. Remarkably, Y223H has not been detected in cancers indicating it is inhibitory to cancer progression. Further analysis showed that most mutations do not affect DGAT2 gene expression suggesting this change is not a major contributor to cancer development. Intriguingly, although most cancers are characterized by low DGAT2 gene expression, some show high expression levels, indicating that, at least in certain cases, over-expression is not inhibitory to cellular proliferation. This work uncovers unknown roles of DGAT2 in cancers and suggests that its function may be more complex than previously appreciated.

